# 

**Published:** 1898-11

**Authors:** 


### BOOKS RECEIVED.

The Treatment of Skin Cancers. By W. S. Gottheil, M. D., Professor of Dermatology at the New York School of Clinical Medicine; Dermatologist to the Lebanon Hospital, the Northwestern and West-side German Dispensaries, etc. Price, $1.00. New York: Published by The International Journal of Surgery, 100 William Street. 1898. (See Book Reviews, this issue.)

The Refraction of the Eye. A Manual for Students. By Gustavus Hartridge, F. R. C. S., Senior Surgeon to the Royal Westminster Ophthalmic Hospital; Ophthalmic Surgeon and Lecturer on Ophthalmic Surgery to the Westminster Hospital; Ophthalmic Surgeon to St. Bartholomew's Hospital, Chatham, etc. With 104 illustrations. Ninth edition. Duodecimo, pp. xvi.—267. London: J. & A. Churchill. Philadelphia: P. Blakiston's Son & Co. 1898.

The Principles and Practice of Hydrotherapy. A Guide to the Application of Water in Disease. For Students and Practitioners of Medicine. By Simon Baruch, M. D., Visiting Physician to the J. Hood Wright (formerly Manhattan General) Hospital; Consulting Physician to the Montifiore Home for Chronic Invalids, etc., etc. With numerous illustrations. Octavo, pp. viii.—435. New York: William Wood & Co. 1898.

A Laboratory Guide to Urinalysis and Toxicology. By R. A. Witthaus, M. D., Professor of Chemistry, Physics and Toxicology in the Medical Department of Cornell University; Professor of Chemistry and Toxicology in the Medical Department, University of Vermont, etc., etc. Fourth edition. New York: William Wood & Co. 1898.

American Pocket Dictionary. Edited by W. A. Newman Dorland, A. M., M. D., Assistant Obstetrician to the Hospital of the University of Pennsylvania; Fellow of the American Academy of Medicine, etc., etc. Containing the pronunciation of over 26,000 words, along with over 60 extensive tables. Philadelphia: W. B. Saunders, 925 Walnut Street.

The Care of the Baby. By J. P. Crozer Griffith, M. D., Clinical Professor of Diseases of Children, University of Pennsylvania. Second edition, revised. Duodecimo, 404 pages, with 67 illustrations in the text and 5 plates. Price, cloth, $1.50. Philadelphia: W. B. Saunders, 925 Walnut Street.

Essentials of Materia Medica, Therapeutics and Prescription Writing, Arranged in Form of Questions and Answers. Especially for Students of Medicine. By Henry Morris, M. D., Physician to St. Joseph's Hospital, etc. Fifth edition, revised and enlarged. Philadelphia: W. B. Saunders, 925 Walnut Street.

Practical Diagnosis. The Use of Symptoms in the Diagnosis of Disease. By Hobart Amory Hare, M. D., Professor of Therapeutics and Materia Medica in the Jefferson Medical College of Philadelphia.. Third edition, enlarged and thoroughly revised. In one 8vo volume of 615 pages, with 204 engravings and 13 full-page colored plates. Cloth, $4.75 net. Philadelphia and New York: Lea Brothers & Co.

The Principles and Practice of Medicine. Designed for the Use of Practitioners and Students of Medicine. By William Osler, M. D., Fellow of the Royal Society; Fellow of the Royal College of Physicians, London; Professor of Medicine in the Johns Hopkins University and Physician-in-Chief of the Johns Hopkins Hospital, Baltimore; Formerly Professor of the Institutes of Medicin

McGill University, Montreal, and Professor of Clinical Medicine in the University of Pennsylvania, Philadelphia. Third edition, entirely revised and enlarged. New York: D. Appleton & Co. 1898.

King's American Dispensatory. By Harvey W. Felter, M. D., Adjunct Professor of Chemistry in the Eclectic Medical Institute, Cincinnati, O.; Co-editor Locke's Materia Medica and Therapeutics; President Ohio State Eclectic Medical Association, etc., etc., and John Uri Lloyd, Ph. M., Professor of Chemistry and Pharmacy in the Eclectic Medical Institute, Cincinnati, O.; Formerly Professor of Pharmacy in the Cincinnati College of Pharmacy; Ex-President of the American Pharmaceutical Association; Author of the Chemistry of Medicines; Drugs and Medicines of North America; Etidorpha; etc., etc. New edition, entirely rewritten and enlarged. Two volume edition, royal 8vo, each volume containing over 950 pages with complete indexes. Cloth, $4.50 per volume, post-paid; sheep, $5.00 per volume, post-paid. Volume I. now ready. Cincinnati, O.: The Ohio Valley Co., Publishers.

A Primer of Psychology and Mental Disease for Use in Training-Schools for Attendants and Nurses and in Medical Classes. By C. B. Burr, M. D., Medical Director of Oak Grove Hospital for Nervous and Mental Diseases, Flint, Mich.; Formerly Medical Superintendent of the Eastern Michigan Asylum; Member of the American Medico-Psychological Association, etc. Second edition, thoroughly revised. 5½ x 7¾ inches. Pp. ix.—116. Extra cloth, $1.00 net. Philadelphia: The F. A. Davis Co., Publishers, 1914–16 Cherry street. New York City: 117 W. Forty-second street. Chicago: 9 Lakeside Building, 218–220 S. Clark street, Chicago, Ill.

Practical Uranalysis and Urinary Diagnosis: A Manual for the Use of Physicians, Surgeons and Students. By Charles W. Purdy, M. D., LL. D. (Queen's University); Fellow of the Royal College of Physicians and Surgeons, Kingston; Professor of Clinical Medicine at the Chicago Post-Graduate Medical School. Author of Bright's Disease and Allied Affections of the Kidneys; also of Diabetes: Its Causes, Symptoms and Treatment. Fourth, revised edition. With numerous illustrations, including photo-engravings and colored plates. In one crown 8vo volume, 365 pages, bound in extra cloth, $2.50 net. Philadelphia: The F. A. Davis Co., Publishers, 1914–16 Cherry street. New York City: 117 W. Forty-second street. Chicago: 9 Lakeside Building, 218–220 S. Clark street.

A Treatise on Diseases of the Ear. Together with a Brief Sketch of the Anatomy and Physiology of this Organ. By Albert H. Buck, M. D., Clinical Professor of the Diseases of the Ear, College of Physicians and Surgeons, Medical Department of Columbia University, New York; Consulting Aural Surgeon, New York Eye and Ear Infirmary and the Presbyterian Hospital. Third revised edition. Octavo, pp. xii.—592. Price, $3.50 net. New York: William Wood & Co. 1898.

Student's Histology. A Course of Normal Histology for Students and Practitioners of Medicine. By Maurice N. Miller, M. D., Late Director of the Department of Normal Histology in Loomis Laboratory, University of the City of New York. Revised by Herbert U. Williams, M. D., Professor of Pathology and Bacteriology, Medical Department University of Buffalo. Third revised edition. Profusely illustrated. One volume, pp. 273. Price, $2.00 net. New York: William Wood & Co. 1898.

Clinical Lectures on Mental Diseases. By Thomas S. Clouston, M. D., Lecturer on Mental Diseases in the University of Edinburgh. New (5th) edition. Crown 8vo, 750 pages, with 19 full-page colored plates. Cloth, $4.25 net. Philadelphia and New York: Lea Brothers & Co., Publishers.

Contributions to Orthopedic Surgery. By A. Sidway Roberts, M. D., Late Surgeon to the Philadelphia Hospital; Orthopedic Surgeon to the Out-Patient Department in the University Hospital; Instructor in Orthopedic Surgery in the University of Pennsylvania; Out-Patient Surgeon to the Episcopal Hospital;

Fellow of the College of Physicians of Philadelphia and of the American Orthopedic Association; Member of the Pennsylvania State Medical Society, Philadelphia County Society, Pathological Society, Neurological Society, etc. With a Brief Biographical Sketch by James K. Young, M. D., Professor of Orthopedic Surgery, Philadelphia Polyclinic; Professor of Orthopedic Surgery, Woman's Medical College, etc. Illustrated, pp. 300. Philadelphia: William J. Dornan, Printer. 1898. From the Editor.

Histology, Normal and Morbid. By Edward K. Dunham, M. D., Professor of General Pathology, Bacteriology and Hygiene, in the University and Bellevue Hospital Medical College, New York. In one very handsome octavo volume of 448 pages, with 363 illustrations. Cloth, $3.25, net. Philadelphia and New York: Lea Brothers & Co., Publishers.

A Text-book of Pathology. By Alfred Stengel, M. D., Instructor in Clinical Medicine in the University of Pennsylvania; Professor of Clinical Medicine in the Woman's Medical College, Physician to the Philadelphia Hospital, Physician to the Children's Hospital, etc. With 372 illustrations. Octavo, pp. 848. Philadelphia: W. B. Saunders. 1898.

Diet and Food. Considered in relation to strength and power of endurance, training and athletics. Hy Alexander Haig, M. A. and M. D. Oxon., F. R. C. P., Physician to the Metropolitan Hospital and the Royal Hospital for Children and Women; Author of Uric Acid as a Factor in the Causation of Disease. With 5 illustrations. Duodecimo, pp. vi.—86. London: J. & A. Churchill, Philadelphia: P. Blakiston's Son & Co. 1898.

Cleft Palate; Treatment of Simple Fractures by Operation; Diseases of Joints; Antrectomy; Hernia, etc.. etc. A series of Clinical Lectures. By W. Arbuthnot Lane, M. S. Duodecimo, pp. 278. From the author. The Medical Publishing Company—Limited (Clinical Journal Office.) 1898.

